# A qualitative analysis of electronic nicotine delivery systems (ENDS) uptake and use among young adult never-smokers in New Zealand

**DOI:** 10.1371/journal.pone.0268449

**Published:** 2022-05-27

**Authors:** Lindsay Robertson, Janet Hoek, Mei-Ling Blank

**Affiliations:** 1 Department of Preventive and Social Medicine, University of Otago, Dunedin, New Zealand; 2 Department of Public Health, University of Otago, Wellington, New Zealand; Medical University of South Carolina, UNITED STATES

## Abstract

**Introduction:**

Electronic nicotine delivery systems (ENDS) likely pose fewer health risks than smoking. Yet ENDS uptake has increased among never-smoking young adults, who likely face greater health risks relative to non-users of ENDS. To date, few qualitative studies have explored ENDS uptake and use by never-smokers.

**Methods:**

We conducted in-depth, semi-structured interviews with 16 current ENDS users from New Zealand aged 18 to 24 years old who reported never having smoked cigarettes regularly. We explored participants’ experimentation with conventional tobacco products, trial, uptake and patterns of ENDS use, and their future intentions regarding both ENDS and conventional tobacco products. We managed the data using NVivo12 and used thematic analysis to interpret the transcripts.

**Results:**

ENDS use enhanced *connection and belonging* by providing communal experiences and facilitating social interactions. Participants’ mastery of tricks generated *social cachet* within friendship groups and counteracted the ENDS-related *stigma* they experienced. Flavours, clouds and devices’ physical attributes provided *stimulation and engagement*, and some used ENDS for stress or appetite *management*. Lastly, participants rationalised ENDS uptake by referencing the far greater risks smoking posed.

**Conclusions:**

ENDS uptake by young adult never-smokers is driven by both psycho-social and functional factors. ENDS provided shared hedonic experiences and physical pleasures, and generated both bonding and bridging social capital, although many participants had also experienced judgement from others for using ENDS. Policies that denormalise ENDS as recreational devices could discourage uptake by never-smokers, though measures will require careful nuancing to avoid deterring smokers from switching to ENDS.

## Introduction

Despite evidence that electronic nicotine delivery systems (ENDS) are less harmful than smoking [1, 2], debate continues over ENDS’ potential population-level impact and uncertainty remains over health risks associated with long-term use [3]. Contentious areas include whether ENDS support smoking cessation or foster dual use (and thus delay cessation) [4–7]; whether ENDS marketing deliberately targets young never-smokers [8, 9]; and whether use by never-smokers may act as a gateway to smoking [10, 11].

Irrespective of ENDS’ potential gateway effects [12, 13], protecting never-smokers from ENDS use is an important public health goal, given ENDS are not risk-free [2, 14]. However, reviews and country-specific studies have found that adolescents and young adults (defined as those under 25 years) may be more likely than adults to trial ENDS [15, 16], and experimentation with ENDS among these groups has increased rapidly in recent years [17, 18]. Similar concerns have arisen in New Zealand (NZ), where in 2018–19, 18–24 year-olds had the highest prevalence of ENDS ever use (47%) and current (i.e., at least monthly) use (9%) [19]. Although ENDS use is positively associated with smoking, many regular ENDS users have never smoked regularly [20, 21], and recent NZ and UK data suggest ENDS use among never-smokers is increasing [22, 23].

Several studies exploring factors that promote ENDS uptake by adolescents or young adults identify curiosity and pleasure in novel flavours as key motivations [20, 22, 24]. Young adults find ENDS attractive and intriguing, and likely to elicit peer approval; ENDS also enable covert nicotine use in smokefree settings [25–27]. Compared to smoked tobacco products, which must often feature standardised packaging, young people report using ENDS as personal fashion accessories [26, 28]. These findings suggest the materiality of ENDS devices enables users to generate social capital in the same way tobacco branding once created a “badge product” that smokers used to represent themselves [29].

Despite strong evidence from surveys outlining why ENDS appeal to young adults, earlier studies do not offer rich, in-depth insights into ENDS uptake and use, and knowledge of how never-smoking young adults incorporate ENDS into their social practices remains limited. Of the qualitative studies exploring factors prompting ENDS uptake, most have either sampled smokers [25, 26, 28, 30–35], or have not reported respondents’ smoking status [26]. One UK qualitative study reported that non-smokers who used ENDS found these offered enjoyable social and recreational experiences [36]. However, that study included only three young adult never-smoking ENDS users and used an open-ended questionnaire, rather than interviews or focus groups that allow detailed probing.

Given motivations for ENDS uptake among never-smokers differ from those of smokers [31], and increasing ENDS use among young people, we examined ENDS uptake among young adult never-smokers to develop a richer understanding of their lived experiences of ENDS use. Identifying factors that promote uptake could help inform measures to reduce experimentation and the risk of a life-long nicotine addiction.

## Methods

### Sample and recruitment

Using social media and community advertising, we recruited participants aged 18 and over from two urban areas in NZ: Dunedin and Wellington. This study formed part of a larger project exploring ENDS uptake and use, in which we used purposeful sampling to maximise demographic diversity in our sample [37], and theoretical sampling as we identified important sub-groups in the sample, including dual users, Māori and Pacific peoples, and young adult never-smokers. Analyses of dual users, and Māori and Pacific peoples are reported elsewhere [38, 39]. We classified participants as never-smokers if they had not smoked 100 cigarettes or more in their lifetime, had never smoked cigarettes regularly (i.e., at least once a month), and had not smoked a cigarette in the past 30 days. We included individuals who self-identified as regular ENDS users (defined as using ENDS at least a couple of times a month). We excluded two never-smokers aged over 24 years to retain a young adult sample and enable an in-depth exploration of that group’s experiences. Participants received a $NZ40 gift voucher to recognise any costs they incurred while participating in the research.

### Data collection

We developed a semi-structured in-depth interview guide that explored specific topics but retained flexible question order and wording to allow detailed probing. To establish rapport and check participants’ smoking status, we first explored past smoking experimentation before probing participants’ trial, uptake, and current ENDS use (S1 and S2 Files contain the interview guide and information sheet). We used time grids to record participants’ ENDS use patterns on an average day and social evening and administered a short questionnaire at the end of the interview to record past smoking, current ENDS use and perceptions, and demographic attributes. All participants gave written informed consent before the interview commenced, and the University of Otago Human Ethics Committee reviewed and approved the study (reference 16/132). We aimed to recruit between 16 to 20 participants; once saturation (defined as no new idea elements in two consecutive interviews) was achieved, data collection stopped. Interviews lasted for approximately 60 minutes and were conducted between January and September 2017.

### Analysis

Interviews were recorded and subsequently transcribed verbatim, and then interpreted using a thematic analysis approach [40]. LR and JH initially coded three transcripts independently using a line-by-line open-coding approach, then met to discuss, compare and refine initial categories, and develop codes. LR and JH subsequently coded a further two transcripts independently using these codes, and conferred to review the initial themes, before confirming these. Using NVivo12, LR coded the remaining transcripts; LR and JH reviewed all coded data, identified, discussed, and confirmed the themes presented. MB reviewed final coding categories reported in the manuscript for coherence and logic.

## Results

### Participants’ characteristics

The sample comprised seven females and nine males, aged between 18 and 24 years old; Table 1 contains participants’ details. All names used are pseudonyms; quotations identify speakers by their ENDS use status (e.g., Violet, daily).

**Table 1:** Participant characteristics.

Pseudonym	Sex	Ethnicity*	ENDS use frequency	Time since first ENDS use	Current e-liquid nicotine level	Device used	Future vaping plans
Abe	M	NZ European	Less than weekly	4 years	1–8 mg	Vape pen	Plans to stop
Ashley^†^	M	Chinese	Less than weekly	18 months	0 mg	Vape pen	Doesn’t know
Bailey	F	Māori	Less than weekly	18 months	Unsure whether e-liquid contains nicotine	Vape pen	Doesn’t know
Cath	F	Chinese	At least once a week	1 year	1–8 mg	Mod/ tank	Plans to stop
Dave	M	NZ European	At least once a week	1 year	0 mg	Mod/ tank	Plans to continue
Gerry^§^	M	Chinese	Daily	15 months	0 mg	Mod/ tank	Plans to continue
Iain^§^	M	NZ European	Daily	9 months	1–8 mg	Mod/ tank	Plans to continue
Jane	F	Other (South African)	Daily	8 months	1–8 mg	Mod/ tank	Doesn’t know
Joseph	M	NZ European	Daily	1 year	1–8 mg	Mod/ tank	Plans to continue
Leonie	F	NZ European	Daily	18 months	1–8 mg	Mod/ tank	Doesn’t know
Libby	F	NZ European	At least once a week	6 months	1–8 mg	Mod/ tank	Plans to continue
Simon	M	NZ European	Daily	4 years	0 mg	Vape pen	Doesn’t know
Tara	F	Māori	At least once a week	1 month	0 mg	Mod/ tank	Doesn’t know
Tom^‡^	M	NZ European	At least once a week	4 months	0 mg	Mod/ tank	Doesn’t know
Violet^‡^	F	Chinese/ European	Daily	1 year	1-8mg	Mod/ tank	Plans to continue
William	M	NZ European	Daily	2 years	0 mg	Mod/ tank	Plans to continue

^*^ Participants could report multiple ethnicities.

^†^ Indicates participant had never tried a conventional cigarette; all other participants had tried smoking but had never smoked regularly (i.e. at least once a week)

^‡^ Indicates participant was educated to Certificate/ Diploma level

^§^ Participant educated to university Bachelor’s degree level; all other participants were educated to high school level

### Thematic analysis

Both psychosocial and functional factors motivated and reinforced participants’ ENDS use. We identified two psychosocial themes (*connection and belonging*, and *balancing social cachet and stigma)* and three functional themes that explained ENDS use (*stimulation and engagement*, *self-management*, and *rationalisations relative to smoking)*.

ENDS use enhanced *connection and belonging* by creating bonding and bridging social capital that facilitated social interactions. Participants’ ability to perform tricks brought recognition and *social cachet*; some gained status as benefactors who shared their device, while others represented themselves through their ENDS device. Yet while ENDS use could enhance participants’ standing among their peers, it could also elicit *stigma* from those outside these groups, who judged never-smokers who used ENDS negatively. Appealing flavours, smells, and clouds *stimulated and engaged* participants, and helped characterise ENDS use as intriguing and enjoyable. A minority used ENDS primarily to *manage* their appetite or alleviate negative affective states, such as anxiety or boredom. Lastly, participants *rationalised* ENDS use by comparing the perceived benefits of ENDS against the disadvantages of smoking. S3 File contains a codebook with quotations that illustrate the themes and sub-themes we developed.

#### Connection and belonging

ENDS uptake typically occurred in social settings. Some participants, such as Violet (daily), wanted to share experiences they observed their peers enjoying, and actively decided to adopt ENDS: *“when a lot of people are doing something around you and it looks like something that’s fun*, *you want to do it as well”*.

Several participants reported ENDS uptake occurred after sharing a friend’s device or being gifted an ENDS; these experiences created bonding social capital and strengthened existing relationships. Participants repaid sharing and gifting experiences by paying forward the goodwill they had received. Abe (< weekly) noted: *“I’d be more than happy to offer them my pen*. *Just like my friend gave it to me*, *um*, *yeah*, *I guess that would be the idea”* and Jane (daily) commented: “*…they all enjoy using my vape as well*. *So they’ll like ask me*, *and then we’ll just pass it around kind of thing…”*.

Bailey (< weekly) and Tom (weekly) elaborated on how ENDS created bonding social capital and used a “circling” metaphor to describe how ENDS moved around a group, piqued interest, and drew people into social groups:

*“…most of the time the e-cigarette sort of circles anyway, because like I said, the tricks thing and they’re new and everyone kinda wants to try them anyway, so they’re just circling…”*.

Whether at a party or in a day-to-day context, ENDS linked people:

*“…everyone seems to pass the thing around all the time*, *if I ever bring it out… they’re all using my one*, *yeah*…*it will just go around in a circle*, *pretty much*. *I don’t get it back for a while (laughs)… everyone’s usually home from uni or finished their day at work or whatever and usually sit in the lounge talking… that’s yeah*, *when everyone’s together*.*”* (Tom, weekly)

As well as reinforcing existing connections, ENDS could also create bridging social capital by providing entry to social groups where participants had previously felt on the periphery; Dave (weekly) explained:

*“…if I was, like, at a bar or something… Um, out with the smokers, sort of thing. At the bar, they’ve got like an outside area. And yeah, quite a few people bring their vapes sort of sticks and pens. Cos there’s people out smoking and sort of like a communal sort of thing*.*”*

As well as connecting participants to ENDS users they had not previously known, ENDS offered access to smoking groups:

*“…Cos you go to a party and*… *it seems to just be something I have in my hand and that I seem to do and everyone’s smoking cigarettes*. *So I don’t know*, *just maybe a ‘fit in’ kind of thing*.*”* (Tom, weekly)

ENDS created and strengthened social connections, and facilitated interactions with others, especially in group situations they had found difficult to join as non-smokers.

#### Balancing social cachet and stigma

Participants enjoyed ENDS’ materiality and used their devices to represent themselves. As personal fashion statements, ENDS evoked attractiveness and sophistication; Jane (daily) had chosen a pink device because she: “*wanted to stick to like a pretty sort of colour*, *nothing too robotic-looking or too mechanical-looking*” while Libby (weekly) stated: “*I’ve got a black one*. *Just because it was a sleek looking kind of thing… it’s just my kind of style*”. Iain (daily) felt buoyed by others’ curiosity: *“I had a couple of people that were a wee bit curious about it and they said*, *‘Oh is that an e-cigarette*?*’…I’m like ‘Yep’*, *and they’re all like*, *‘Sick*!*’”*.

ENDS became performance props; participants integrated these into practices where they created social cachet that reinforced on-going use. William (daily) explained:

*“…it was purely because of the sort of skills and tricks you can do with it*. *That’s still sort of why I only do it*, *is to sort of get the tricks and stuff*. *My friends are always really impressed when I do something like that*. *Like if I bring out the vape*, *they’re all like*, *‘[sigh] really*?*’ But then if I do some tricks*, *they’re all like*, *‘Yo*, *that’s pretty cool*!*’”*.

William’s comments hint at the ambivalence surrounding ENDS; his friends “sigh” when he brings out his device, but their indifference turns to admiration as he becomes not merely an ENDS user, but a performer. Bailey (< weekly) commented on the status performers enjoyed: *“it’s cool to see if someone can pull them off and you can always tell the people who already know how to do it…the people who can do it*, *the group’s reaction is excitement*, *and ‘Oh*, *that was really good*! *That was cool*!*’”*.

Although performing vape tricks elicited peer approval, several participants chose not to vape in public as they sensed judgment from those outside their social circle. Not having dealt with the stigma of smoking, negative user stereotypes presented unwelcome challenges, as Jane (daily) explained: *“*…*if I’m having a vape*, *they will come up to me and like say*, *"Oh*, *Vape Naysh*.*"*… . *Yeah*… . *I don’t take offence to it or anything*, *but*, *yeah*, *um*. *Yeah*, *I think if some*… *if I was just walking down the street*, *I do get a little bit like*, *"Oh*, *what if someone’s judging me*,*" or*, *yeah*… . *maybe just sort of laughing at me or something*.*”*.

Some attributed perceived judgment to their non-smoking status; unlike smokers who switched to ENDS and gained respect, non-smokers appeared contradictory and perplexing, as William (daily) noted:

*“…cause it’s quite well known that smoking is extremely bad for you, um, and if you make the effort to get off smoking and get onto vaping*, *you get a lot more respect for it… compared to people who just do it for the sake of it… there’s not much respect in that…”*

While participants enjoyed performing to their peers, their ostentation had limits and they differentiated themselves from ENDS-user communities, which Iain described as “*a wee bit obnoxious*… *a wee bit try hardy*… *a little bit too showy offy*.*”*

#### Stimulation and engagement

Nearly all participants enjoyed e-liquid flavours (despite some experiences of unpleasant flavours); many had experimented with ENDS because intriguing aromas had aroused their interest. Violet (daily) explained: *It just has a really nice smell which is*, *I think*, *the reason why people want to try it because it just smells so good*.*”*

Most preferred fruity or sweet e-liquids, including *“lollies”* or *“candy”*, such as *“Starburst”* (Simon, daily) or *“Cola Chuppa Chups”* (Libby, weekly), rather than tobacco flavours, which they saw as targeted to smokers and thus not relevant to them. Flavour variety maintained their interest in ENDS; Violet (daily) felt that *“*…*where you only have one flavour*, *um*, *after a while you get desensitised to it*, *and you don’t taste it anymore”*. William (daily) also explained how diverse flavours kept him engaged:

*“… there’s lots of flavours out there, which I’m looking to try… lots of blends of fruits and menthol and everything… there’s lots of, like, really interesting flavours, like custard pie and there’s a Turkish delight one, which looks really nice, and*, *um…just all these different desserts you can get…”*

Clouds provided a different enjoyment; their visual novelty and embodiment of breath intrigued participants, as Gerry (daily) explained: *“I guess it was quite cool*… *just seeing the vapour being exhaled from your mouth*. *It’s just something you don’t really see often*. *The effect*, *I suppose*. *The visual effect”*. Tara (weekly) also found clouds’ volume and density appealing: *“…just how dense the steam was*, *it looks*, *I just think it looks really cool*, *I guess just because there’s so much of it”*. Participants used “fantastical” clouds creatively and some felt drawn to cloud chasing communities they had seen on social media:

*“…you can just*, *um*, *either breathe it into a cup and then it stays in the cup and you can play with it*.. *I feel like I sound like*, *kind of like*, *a five year old*, *but that is the most appealing thing I can think about it*.*”* (Violet, daily)

*“… I don’t know it might seem interesting or fantastical or whatever*, *um*, *just sort of the dragon analogy*. *I don’t know*. *It’s just fun to see like a cloud that you make*.*”* (Leonie, daily)

A minority focussed on device customisation as a hobby. Iain (daily) enjoyed *“playing around”* with his device and enjoyed the control he could exert over his experiences:

*“…I really like fiddling with things and taking things apart and playing around with it and all that*, *and this one’s got a tonne more options for how much control I can have over the experience… and I really like having the customisation abilities…”*

Joseph (daily) also gained tactile pleasure from the hand-feel his device afforded:

*“*…*I quite liked the*, *kinda having a square box rather than*, *you know*, *something that looked like a pen*.…*something that you could really feel*, *holding it in your hands*, *rather than you know*, *trying to bring a pencil to my face*.*”* (Joseph, daily).

#### Self-management

Several participants used ENDS to help manage stress; Cath (weekly) explained: *“I would say during*, *like when I’m stressed*, *I tend to vape more*. *So*… *during exam time”*. Inhaling and exhaling deeply offered a moment to pause; Violet (daily) explained: *“it’s something that’s kind of forcing you to just sort of sit there and breathe”*. For Simon (daily), ENDS use enabled him to step back, take stock, and assess a situation before continuing:

*“…when you’re obviously breathing in something and you’re kind of just taking a step back from something*. *Like, when I’m doing my study…it’s kind of nice just to kind of sit back and be like, ‘Okay’, like breathe in, take it like the vape and have a taste, and then just be like… ‘Here we go. This is what I need to be doing.’”*

As well as providing a pause, ENDS helped participants manage anxiety or boredom in social situations; Tom (weekly) explained: *“It was like something that just keeps you busy when you’re in a crowd and stops you from getting*, *I don’t know*, *bored and awkward”*.

A minority used ENDS to regulate their eating or energy drink consumption, and as an alternative to comfort eating during stressful times. Gerry (daily) found using sweet flavoured e-liquid was *“just like when you’re stressed and you get lollies [confectionery]*, *it’s kind of like that…”*. Similarly, Violet (daily) explained that: *“…like*, *if I stress eat*, *this is something I can do instead of stress eating*…*”* One participant reported purchasing an ENDS and commencing daily use after reading online that ENDS could help people “*eat less”*:

*“*…*probably like maybe two times a day*, *if I’m feeling a little bit hungry but I’ve already eaten… kind of like that*, *it’s my thing to go to and I just have something in my mouth type thing…”* (Simon, daily).

However, despite participants’ stated intentions, ENDS’ role in managing food or drink intake was less clear. For instance, although Simon (daily) reported eating “*a lot less*” since he started using an ENDS, he also felt his device could be *“a placebo”*.

#### Rationalisations relative to smoking

Participants believed ENDS offered multiple ‘benefits’, such as pleasure, connections, and social cachet, and had few costs compared to smoking, which they viewed as “bad” and guilt-inducing:

“*I kind of just made a decision*, *like it doesn’t seem anywhere near as bad as cigarettes*, *regardless of how bad it is anyway*, *so I think I’ll be alright with it*…*”* (Iain, daily)

Associating ENDS with more benefits than costs privileged ENDS use over smoking: *“…smoking didn’t appeal because it’s like*… *there’s not a lot of benefit that you get from it versus what the harms are*, *for me personally*.*”* (Violet, daily) Participants’ implicit harm hierarchy enabled them to avoid the self-excoriation and guilt associated with smoking, as Iain set out: *“…I feel less guilty about using it ‘cos I remember even on the odd occasion I would have a cigarette*, *I always felt pretty dumb after it*, *but with vaping*, *I just*, *I don’t feel that guilt and it’s still just fun for me*.*”*

In addition to weighing up potential long-term health risks, participants found ENDS helped them avoid unpleasant physical sensations of smoking, including the bad odour and taste, harsh throat-hit, and nausea:

*“…after vaping it would just be*, *like*, *I would feel relaxed and it would just kind of fade off*. *Whereas with cigarettes it would be*, *like…a sick feeling*.*”* (Cath, weekly)

Guilt associated with smoking even an occasional cigarette diminished the experience beyond the unpleasant physical effects some participants reported. By contrast, ENDS offered a conduit to relaxation and pleasure, and avoided the physical and metaphorical taint of smoking.

## Discussion

A complex array of psycho-social and functional factors prompted ENDS use among these never-smoking participants. ENDS use created bonding and bridging social capital; it connected them to social groups, drew them into communities, and provided gratifying status and attention. Beyond bonding, participants gained pleasure from ENDS, whether through flavours, clouds or via the device itself, and increased control over stress and appetites, while bringing none of the physical or social risks they associated with smoking.

We extend earlier work by explaining how ENDS foster communal activities that enable non-smokers to “belong” and manage social anxieties, which many young people experience as they transition from adolescence to adulthood [26, 32, 41]. Participants used ENDS as materials in social practices where they showcased their competencies and created social capital [29]; gaining esteem as skilled community members may contribute to young adults’ self-actualisation [42, 43]. Several used ENDS as part of their “extended-self”, to signal valued identity attributes to others and reinforce those same attributes to themselves [44]. Though our data were collected prior to the COVID-19 pandemic, the widespread sharing of ENDS among young adults has clear implications for infectious disease transmission and could help explain a recently reported association between ENDS use and positive COVID-19 status among U.S. youth [45].

A minority reported using ENDS flavours as a substitute for consuming food or confectionery, behaviours they wished to control more effectively, particularly during times of stress. While UK data suggest perceptions of ENDS as weight control tools motivate comparatively few ENDS users [46], a recent U.S. study indicate anxiety relief may be more important to never-smokers than to former or current smokers [47]. Our findings suggest further research is needed to explore never-smokers’ perceptions of ENDS as a self-management tool and to examine factors (e.g. gender, nicotine use) associated with initiating ENDS for weight or stress management.

Whether they privileged the diverse flavour range that stimulated on-going interest, the embodied breath that clouds represented, or the technical sophistication of their device and the mastery they could display, ENDS created enjoyment. We extend earlier studies that found these attributes support experimentation with ENDS by young never-smokers [31, 33, 41, 48–50], and explain how ENDS contribute to hedonic experiences and create social cachet.

Put in the context of population studies documenting rising ENDS use among never-smokers, our findings suggest policy makers may need to introduce additional measures, if they are to balance unwanted ENDS uptake among never-smokers against movement by smokers to a less harmful option. Measures such as limiting e-liquid flavours and flavour descriptions to generic terms, such as “fruit-citrus” or “candy”, could reduce never-smokers’ interest in ENDS use [25, 31, 51, 52]. New Zealand has recently limited sales of diverse flavours to R18 (i.e. adult only) specialist ENDS stores, in an attempt to reduce ENDS uptake among never-smokers [53]. Limiting e-liquid flavours and regulating the propylene glycol and vegetable glycerin ratio to reduce the cloud volume produced could further reduce ENDS’ appeal to youth and young adults. Restricting marketing that positions ENDS as recreational devices (rather than potential harm reduction tools) could also reduce exposure to appeals that encourage experimentation and regular use [9].

Like all studies, our work has limitations. Although our findings provide rich insights into ENDS uptake motivations and continuing use, our sample of 16 ENDS users means we cannot generalise our findings to the wider population of young adults. Because we collected the data in 2017, recent product developments–such as the introduction of pod devices like Juul and Vuse, disposable ‘puff bars’, and products using high concentration nicotine salts–may have affected ENDS users’ motivations and experiences in ways that we have not captured. Around half of our participants reported using nicotine-free e-liquids, suggesting that obtaining nicotine was not a main reason for ENDS use in our sample; however, recent data suggest the vast majority of young daily and weekly ENDS users consume nicotine [54]. Nicotine dependence may thus have emerged as an important issue experienced by never-smokers since we collected our data. Lastly, we cannot necessarily assume our findings are generalisable to youth. The main reasons for youth vaping include experimentation (i.e. to “see what it’s like”), entertainment, and enjoyable flavours [e.g. 55, 56]. While further studies could help address youth vaping by examining psychosocial contributors to uptake among that population group, our study may still provide relevant insights as to how youth experimentation develops into more regular use [57]. Notwithstanding these limitations, our study provides novel data on an understudied topic in an important population group. Further, it highlights the predominantly psychosocial factors that contribute to ENDS uptake by young adults; from a developmental perspective, these are unlikely to have changed substantially in different cohorts of young adults. Experiences of stigma associated with ENDS use could have changed as social norms evolve; this question warrants further investigation. However, participants’ experiences with flavours, clouds and “tinkering” appear to have continuing relevance, despite the emergence of new products [18]. Further, the lack of qualitative research with never-smokers who use ENDS, together with evidence of increasing experimentation among this group, means our study creates a platform on which future work may build.

Overall, our findings mirror evidence from historical tobacco industry documents, which reveal that smoking typically starts in social settings, evolves into a habitual practice to manage stress or other negative psychological states, and then becomes an addiction [58]. Just as reducing combusted tobacco use among young people required comprehensive measures that spanned community-based initiatives to policy interventions, so reducing ENDS uptake among never-smokers will likely require diverse initiatives. Measures that have successfully denormalised conventional tobacco products, including taxation, decreasing retail visibility, warning labels, and smoke-free policies [59, 60], represent important starting points. Nonetheless, these measures will require careful nuancing to avoid deterring smokers unable to quit using other methods from switching to ENDS.

## Supporting information

S1 File Interview protocol. (PDF)

S2 File Participant information sheet. (PDF)

S3 File Codebook. (PDF)

## References

[1] McNeill A., et al. E-cigarettes: an evidence update. A report commissioned by Public Health England. 2015. 2015 12 March 2020]; Available from: www.gov.uk//government/uploads/system/uploads/attachment_data/file/457102/Ecigarettes_an_evidence_update_A_report_commissioned_by_Public_Health_England_FINAL.pdf.

[2] U.S. National Academies of Science Engineering and Medicine. Public Health Consequences of E-Cigarettes. 2018 12 March 2020]; Available from: http://nationalacademies.org/hmd/Reports/2018/public-health-consequences-of-e-cigarettes.aspx. 29894118

[3] Marques P., Piqueras L., and Sanz M.-J., An updated overview of e-cigarette impact on human health. Respiratory Research, 2021. 22(1): p. 1–14. 34006276 10.1186/s12931-021-01737-5 PMC8129966

[4] Owusu D., et al., Patterns and trends of dual use of e-cigarettes and cigarettes among US adults, 2015–2018. Preventive medicine reports, 2019. 16: p. 101009. doi: 10.1016/j.pmedr.2019.101009 31763161 PMC6861646

[5] El Dib R., et al., Electronic nicotine delivery systems and/or electronic non-nicotine delivery systems for tobacco smoking cessation or reduction: a systematic review and meta-analysis. BMJ open, 2017. 7(2). 10.1136/bmjopen-2016-012680 PMC5337697 28235965

[6] Patil S., et al., Are electronic nicotine delivery systems (ENDs) helping cigarette smokers quit?—Current evidence. Journal of Oral Pathology & Medicine, 2020. 49(3): p. 181–189. doi: 10.1111/jop.12966 31642553

[7] Brown J., et al., Real‐world effectiveness of e‐cigarettes when used to aid smoking cessation: a cross‐sectional population study. Addiction, 2014. 109(9): p. 1531–1540. doi: 10.1111/add.12623 24846453 PMC4171752

[8] Padon A.A., Maloney E.K., and Cappella J.N., Youth-targeted e-cigarette marketing in the US. Tobacco regulatory science, 2017. 3(1): p. 95–101. doi: 10.18001/TRS.3.1.9 28083545 PMC5221880

[9] Hoek J. and Freeman B., BAT(NZ) draws on cigarette marketing tactics to launch Vype in New Zealand. Tobacco Control, 2019. 28(e2): p. tobaccocontrol-2019-054967. doi: 10.1136/tobaccocontrol-2019-054967 31315965

[10] Khouja J.N., et al., Is e-cigarette use in non-smoking young adults associated with later smoking? A systematic review and meta-analysis. Tobacco Control, 2020. doi: 10.1136/tobaccocontrol-2019-055433 32156694 PMC7803902

[11] Soneji S., et al., Association between initial use of e-cigarettes and subsequent cigarette smoking among adolescents and young adults: a systematic review and meta-analysis. JAMA pediatrics, 2017. 171(8): p. 788–797. doi: 10.1001/jamapediatrics.2017.1488 28654986 PMC5656237

[12] Kim S. and Selya A.S., The relationship between electronic cigarette use and conventional cigarette smoking is largely attributable to shared risk factors. Nicotine & Tobacco Research, 2020. 22(7): p. 1123–1130. doi: 10.1093/ntr/ntz157 31680169 PMC7291806

[13] Etter J.F., Gateway effects and electronic cigarettes. Addiction, 2018. 113(10): p. 1776–1783. doi: 10.1111/add.13924 28786147

[14] Wise J., E-cigarettes are safer than smoking but not without risks, concludes toxicity review. BMJ 2020. 370: p. m3529. doi: 10.1136/bmj.m3529 32912929

[15] Reid J.L., et al., Who is using e-cigarettes in Canada? Nationally representative data on the prevalence of e-cigarette use among Canadians. Preventive Medicine, 2015. 81: p. 180–183. doi: 10.1016/j.ypmed.2015.08.019 26348453

[16] Levy D.T., Yuan Z., and Li Y., The prevalence and characteristics of e-cigarette users in the US. International journal of environmental research and public health, 2017. 14(10): p. 1200. 10.3390/ijerph14101200 PMC5664701 29019917

[17] Cullen K.A., et al., Notes from the field: use of electronic cigarettes and any tobacco product among middle and high school students—United States, 2011–2018. Morbidity and Mortality Weekly Report, 2018. 67(45): p. 1276. doi: 10.15585/mmwr.mm6745a5 30439875 PMC6290807

[18] Fadus M.C., Smith T.T., and Squeglia L.M., The rise of e-cigarettes, pod mod devices, and JUUL among youth: factors influencing use, health implications, and downstream effects. Drug and alcohol dependence, 2019. 201: p. 85–93. doi: 10.1016/j.drugalcdep.2019.04.011 31200279 PMC7183384

[19] New Zealand Ministry of Health. New Zealand Health Survey: Annual Data Explorer—Tobacco use. 2019 30 September 2020]; Available from: https://minhealthnz.shinyapps.io/nz-health-survey-2018-19-annual-data-explorer/_w_9f9a847c/#!/home.

[20] Wamamili B., et al., Electronic cigarette use among university students aged 18–24 years in New Zealand: Results of a 2018 national cross-sectional survey. BMJ Open, 2020. 10(6). 10.1136/bmjopen-2019-035093 PMC7311043 32571858

[21] ASH NZ. 2019 ASH Year 10 Snapshot: Vaping and E-cigarettes. 2019 30 September 2020]; Available from: https://d3n8a8pro7vhmx.cloudfront.net/ashnz/pages/70/attachments/original/1583197938/2019_ASH_Y10_Snapshot_E-cigs_and_vaping_FINAL.pdf?1583197938.

[22] ASH UK, Use of E-cigarettes Among Adults in Great Britain 2019. 2019, ASH UK: London.

[23] Lucas N., Gurram N., and Thimasarn-Anwar T. Smoking and vaping behaviours among 14 and 15-year-olds: results from the 2018 Youth Insights Survey. 2020 12 October 2020]; Available from: https://www.hpa.org.nz/sites/default/files/Smoking%20and%20vaping%20behaviours%20among%2014%20and%2015-year-olds%20report2.pdf.

[24] Schneller L.M., et al., Use of flavored e-cigarettes and the type of e-cigarette devices used among adults and youth in the US—Results from wave 3 of the population assessment of tobacco and health study (2015–2016). International Journal of Environmental Research and Public Health, 2019. 16(16): p. 2991. 10.3390/ijerph16162991 PMC6720922 31434229

[25] Choi K., et al., Young adults’ favorable perceptions of snus, dissolvable tobacco products, and electronic cigarettes: findings from a focus group study. American journal of public health, 2012. 102(11): p. 2088–2093. doi: 10.2105/AJPH.2011.300525 22813086 PMC3469759

[26] Pokhrel P., et al., Young adult e-cigarette users’ reasons for liking and not liking e-cigarettes: a qualitative study. Psychology & health, 2015. 30(12): p. 1450–1469. doi: 10.1080/08870446.2015.1061129 26074148 PMC4657726

[27] Brown R., et al., A qualitative study of e-cigarette emergence and the potential for renormalisation of smoking in UK youth. International Journal of Drug Policy, 2020. 75. doi: 10.1016/j.drugpo.2019.11.006 31785547 PMC6983925

[28] McDonald E.A. and Ling P.M., One of several ‘toys’ for smoking: young adult experiences with electronic cigarettes in New York City. Tobacco control, 2015. 24(6): p. 588–593. doi: 10.1136/tobaccocontrol-2014-051743 25564287 PMC4492884

[29] Blue S., et al., Theories of practice and public health: understanding (un) healthy practices. Critical Public Health, 2016. 26(1): p. 36–50.

[30] Han G. and Son H., What influences adolescents to continuously use e-cigarettes? Public Health Nursing, 2020. 37(4): p. 504–509. doi: 10.1111/phn.12735 32372518

[31] Berg C.J., Preferred flavors and reasons for e-cigarette use and discontinued use among never, current, and former smokers. International journal of public health, 2016. 61(2): p. 225–236. doi: 10.1007/s00038-015-0764-x 26582009 PMC4808473

[32] Hoek J., Thrul J., and Ling P., Qualitative analysis of young adult ENDS users’ expectations and experiences. BMJ open, 2017. 7(3). doi: 10.1136/bmjopen-2016-014990 28270392 PMC5353280

[33] Tokle R. and Pedersen W., “Cloud chasers” and “substitutes”: e-cigarettes, vaping subcultures and vaper identities. Sociology of Health and Illness, 2019. 41(5): p. 917–932. doi: 10.1111/1467-9566.12854 30677161

[34] Yule J.A. and Tinson J.S., Youth and the sociability of “Vaping”. Journal of Consumer Behaviour, 2017. 16(1): p. 3–14.

[35] Lucherini M., Rooke C., and Amos A., “They’re thinking, well it’s not as bad, I probably won’t get addicted to that. But it’s still got the nicotine in it, so…”: Maturity, Control, and Socializing: Negotiating Identities in Relation to Smoking and Vaping—A Qualitative Study of Young Adults in Scotland. Nicotine & Tobacco Research, 2019. 21(1): p. 81–87. 29126149 10.1093/ntr/ntx245 PMC6302351

[36] Wilson G.L., et al., A thematic analysis of smokers’ and non-smokers’ accounts of E-cigarettes. Journal of Health Psychology, 2020. 10.1177/1359105320909877 PMC8739557 32131638

[37] Patton M.Q., Qualitative research & evaluation methods: Integrating theory and practice. 2014, Thousand Oaks, CA: Sage Publications.

[38] Robertson L., et al., Dual use of electronic nicotine delivery systems (ENDS) and smoked tobacco: a qualitative analysis. Tobacco control, 2019. 28(1): p. 13–19. doi: 10.1136/tobaccocontrol-2017-054070 29419488 PMC6317506

[39] Strickett E., et al., A qualitative analysis of Māori and Pacific people’s experiences of using electronic nicotine delivery systems (ENDS). Nicotine & Tobacco Research, 2021. 23(3): p. 550–556. doi: 10.1093/ntr/ntaa087 32421174

[40] Braun V. and Clarke V., Thematic analysis, in APA handbooks in psychology®. APA handbook of research methods in psychology, Vol. 2. Research designs: Quantitative, qualitative, neuropsychological, and biological, Cooper H., et al., Editors. 2012, American Psychological Association.

[41] Measham F., O’Brien K., and Turnbull G., “Skittles & Red Bull is my favourite flavour”: E-cigarettes, smoking, vaping and the changing landscape of nicotine consumption amongst British teenagers–implications for the normalisation debate. Drugs: Education, Prevention and Policy, 2016. 23(3): p. 224–237.

[42] Martino J., Pegg J., and Frates E.P., The Connection Prescription: Using the Power of Social Interactions and the Deep Desire for Connectedness to Empower Health and Wellness. American Journal of Lifestyle Medicine, 2015. 11(6): p. 466–475. doi: 10.1177/1559827615608788 30202372 PMC6125010

[43] Maslow A.H., Motivation and personality. 1981: Prabhat Prakashan.

[44] Belk R., Possessions and the extended self. Journal of Consumer Research, 1988. 15.

[45] Gaiha S.M., Cheng J., and Halpern-Felsher B., Association between youth smoking, electronic cigarette use, and COVID-19. Journal of adolescent health, 2020. 67(4): p. 519–523. 10.1016/j.jadohealth.2020.07.002 PMC7417895 32798097

[46] Jackson S.E., et al., Vaping for weight control: A cross-sectional population study in England. Addictive behaviors, 2019. 95: p. 211–219. doi: 10.1016/j.addbeh.2019.04.007 30981033 PMC6555398

[47] Al-Hamdani M., et al., Perceptions and Experiences of Vaping Among Youth and Young Adult E-Cigarette Users: Considering Age, Gender, and Tobacco Use. Journal of Adolescent Health, 2020(10.1016/j.jadohealth.2020.08.004). 32943292

[48] Harrell M.B., et al., Flavored e-cigarette use: Characterizing youth, young adult, and adult users. Preventive medicine reports, 2017. 5: p. 33–40. doi: 10.1016/j.pmedr.2016.11.001 27896041 PMC5121224

[49] Leventhal A.M., et al., Effects of non-tobacco flavors and nicotine on e-cigarette product appeal among young adult never, former, and current smokers. Drug and Alcohol Dependence, 2019. 203: p. 99–106. doi: 10.1016/j.drugalcdep.2019.05.020 31434028 PMC7489757

[50] Schneller L.M., et al., Use of flavored electronic cigarette refill liquids among adults and youth in the US—Results from Wave 2 of the Population Assessment of Tobacco and Health Study (2014–2015). PloS one, 2018. 13(8): p. e0202744. doi: 10.1371/journal.pone.0202744 30138412 PMC6107209

[51] Havermans A., et al., Nearly 20 000 e-liquids and 250 unique flavour descriptions: An overview of the Dutch market based on information from manufacturers. Tobacco Control, 2019. doi: 10.1136/tobaccocontrol-2019-055303 31685584 PMC7803909

[52] Soule E.K., et al., Content analysis of internet marketing strategies used to promote flavored electronic cigarettes. Addictive behaviors, 2019. 91: p. 128–135. doi: 10.1016/j.addbeh.2018.11.012 30606627

[53] New Zealand Parliament, Smokefree Environments and Regulated Products (Vaping) Amendment Bill. 2020. 10.1136/tobaccocontrol-2021-057123 35228319

[54] Ball J., et al., New Zealand Youth19 survey: vaping has wider appeal than smoking in secondary school students, and most use nicotine-containing e-cigarettes. Australian and New Zealand Journal of Public Health, 2021. 45(6): p. 546–553. doi: 10.1111/1753-6405.13169 34648227

[55] Evans-Polce R.J., et al., Reasons for vaping among US 12th graders. Journal of Adolescent Health, 2018. 62(4): p. 457–462. 10.1016/j.jadohealth.2017.10.009 PMC5866738 29273302

[56] Gilley M. and Beno S., Vaping implications for children and youth. Current Opinion in Pediatrics, 2020. 32(3): p. 343–348. doi: 10.1097/MOP.0000000000000889 32332326

[57] Villanti A.C., et al., Preventing smoking progression in young adults: the concept of prevescalation. Prevention Science, 2019. 20(3): p. 377–384. doi: 10.1007/s11121-018-0880-y 29525899 PMC6131072

[58] Ling P.M. and Glantz S.A., Why and how the tobacco industry sells cigarettes to young adults: evidence from industry documents. American journal of public health, 2002. 92(6): p. 908–916. doi: 10.2105/ajph.92.6.908 12036776 PMC1447481

[59] Anderson P. and Hughes J.R., Policy interventions to reduce the harm from smoking. Addiction, 2000. 95(1s1): p. 9–11. doi: 10.1080/09652140032017 10723816

[60] Morris D.S., Fiala S.C., and Pawlak R., Opportunities for policy interventions to reduce youth hookah smoking in the United States. Preventing Chronic Disease, 2012. 9(e165), doi: 10.5888/pcd9.120082 23153772 PMC3505114

