# Supplementary material for: A qualitative analysis of electronic nicotine delivery systems (ENDS) uptake and use among young adult never-smokers in New Zealand
Source: PLoS One. 2022 May 27;17(5):e0268449. doi: 10.1371/journal.pone.0268449 (PMC9140280; doi:10.1371/journal.pone.0268449)
Supplement: S1 File — (PDF) [file pone.0268449.s001.pdf]

INTERVIEW PROTOCOL: PHASE ONE

Supporting informed e-cigarette use: A mixed methods study

Non-smoker version

Introduction (Five minutes – ensure participant has IS in advance)
Hello I'm [interviewer name]; I arranged to meet with you to talk about the research I'm doing into e-cigarettes and smoking. Before we start I'd like to show you some information about the interview and check to see whether you have any questions about my work. Here's another copy of the information sheet you were sent when you indicated you were interested in the project; please take a few moments to look through this sheet.

- **PROVIDE PARTICIPANT WITH AN INFORMATION SHEET AND OUTLINE THE KEY POINTS IN THIS. ALLOW TIME TO READ THE INFORMATION SHEET.**

Do you have any questions about the study?

- **EXPLAIN RECORDING OF THE INTERVIEW AND PARTICIPANT'S RIGHTS IN RELATION TO THIS. ONCE PARTICIPANT HAS AGREED TO INTERVIEW BEING RECORDED, TURN RECORDER ON AND NOTE THAT IT IS NOW ON.**

READ OUT WHILE RECORDER IS ON:

As a participant in the research, you have the right to ask questions at any time, to decide if you would prefer not to answer some questions, to receive a copy of the findings, and to withdraw from the research at any time. Please note that your responses will be confidential to the research team members.

- **CHECK AGAIN WHETHER PARTICIPANT HAS ANY QUESTIONS ABOUT THE INTERVIEW.**
- **IF NO QUESTIONS, ASK THE PARTICIPANT TO SIGN AND DATE THE CONSENT FORM.**

Smoking History (Five minutes – just an introduction)
I'd like to start by asking you some questions about smoking:

1. Have you ever tried smoking tobacco? Can you tell me a bit about what happened? **Probe: How old were you? Where did you get the cigarettes from? Who were you with? Where were you? What do you remember about that first experience of smoking?**
2. And what happened after those first cigarettes? **Probe: When did you next smoke? Can you describe your smoking patterns for me? About how many cigarettes were you smoking? When did you normally smoke? Were there any times when you might smoke more or less during the day? Do you ever use other forms of tobacco, like shisha or waterpipe tobacco? Tell me about your use of these types of tobacco. Probe: Explore increased smoking in social settings and when drinking.**

Smoking Day and Night Grids (Again, five minutes timing)
ONLY ADMINISTER IF PARTICIPANT REPORTS CURRENT SMOKING

3. Could you look at this form please [**GIVE RESPONDENT THE SMOKING WEEKDAY GRID HERE].** It has rows that show each hour of the day. I'd like you to think about an **average weekday day** and write in what you're doing and when you are smoking please. Please take as much time as you need to fill it in.

4. Now here's a similar night time grid [**GIVE RESPONDENT THE SMOKING SOCIAL NIGHT GRID HERE].** This time I'd like you to think about evenings when you're out socialising and when you smoke then.

E-cigarette Uptake (Ten minutes, probe information (Q7 and 8 particularly)

5. Now I'd like to ask you about e-cigarettes. When did you first see an e-cigarette being used? And when did you first use an e-cigarette? Who were you with? Was it your own e-cigarette? **Probe: if not, where did you get the e-cigarette from?**

6. What type of EC was it? **[Show images of different EC options, including first and second and third generation e-cigarettes).** Do you know if it had nicotine? Do you remember what flavour it was? What was that first experience like? How did it compare to smoking?

7. What got you thinking about using an e-cigarette more regularly? What kind of information did you want to know about them? Where did you try to find this information? **Probe: explore all sources of information, including websites, vape stores, and word of mouth. Where did you look? What did you find? What did it say? How helpful was that information?**

8. Have you seen e-cigarettes advertised? **What did the advertising say? What did you think of those claims? Probe: Explore benefits such as reduced harm, help quitting among existing smokers.**

E-Cigarette On-going Use (Five to ten minutes; particular probing of Q10)

9. When did you start using an e-cigarette more regularly? What sort did you use? Did it have nicotine? How did you find using it? Have you tried any others? Did the other ECs you have tried have nicotine? **Probe: Explore history of e-cigarette use. [Show images of different EC options, including first and second and third generation e-cigarettes)**.

10. **[Where relevant]** What led you to move from one e-cigarette to another? **How did you find out about different options? Where did you get information from? For each source mentioned, probe what details were ascertained and how useful these were.**

11. What sort of e-cigarette are you using now? Is this your own EC? What made you decide to buy this particular EC? Do you recall how much it cost? **Probe: What features did it have? What made these important to you? If multiple ECs used, probe when different devices used.**

12. Does your EC or vaporiser have nicotine? What about the flavours you use, what are they? What sort of PG /VG mix do you use? What is it about this mix that you like? **How long have you been using this e-cigarette? How do you find it?**

E-Cigarette Day and Night Grids (Five minutes)

13. Could you look at this form please [**GIVE RESPONDENT THE E-CIGARETTE WEEK DAY GRID HERE].** It has rows that show each hour of the day and I'd like you to think about an **average weekday day** and write in what you're doing and when you are using it please. Please take as much time as you need to fill it in.

14. Now here's a similar night time grid [**GIVE RESPONDENT THE E-CIGARETTE SOCIAL NIGHT GRID HERE].** This time I'd like you to think about evenings when you're out socialising and when you use your e-cigarette smoke then.

E-Cigarette and Smoking Use Patterns (Five to ten minutes)
ONLY IF PARTICIPANTS ARE ALSO SMOKING

15. Are you smoking tobacco as well as vaping? **REVIEW DAY GRIDS HERE** Let's look at a normal day and when you vape and smoke? **Probe – can you tell me why you smoke or vape at these times? Are you just smoking or vaping, or both? REVIEW NIGHT GRIDS HERE** And what about when you are out socialising, when are you smoking and vaping?

16. About how long have you been smoking and vaping? Just thinking about an average day **[summarise charts]** about how many times a day would you smoke? And how often would you vape each day? **Probe: Is this a stable pattern of smoking and vaping, or has it changed over time (If yes, probe how it has changed).**

17. Now thinking about a night when you are out socialising, about how many times a night when you're out would you smoke? And how often would you vape? **Probe: Is this a stable pattern of smoking and vaping, or has it changed over time (If yes, probe how it has changed).**

18. Overall, how do you see your smoking and vaping developing? **Probe: Would you like to stop smoking completely? IF YES: What is it that keeps you smoking instead of just vaping? IF NO: What got you vaping if you don't want to stop smoking?**

19. If you're just vaping now, how often do you vape compared to how often you used to smoke? **Probe differences – can you tell me a bit about these differences**?

Experiences of EC Use and Perceived Risks and Benefits (Ten minutes – an important section)

20. **If participants have smoked before.** How does the experience of vaping compare to smoking? **Probe, which did they prefer (check throat hit, light head feelings and whether these were present or missed).** What are the pros and cons compared to smoking tobacco? If someone asked you the best thing about vaping, what would you say? Is there anything you miss about smoking? **Probe source and basis of information.**

21. How do you think the potential harm of using e-cigarettes compares to the potential harm from smoking? What makes you think that? **Probe source and basis of information; probe specific details where possible.**

22. What about for people who are using e-cigarettes as well as smoking? Do you think they face any potential harm? What would that be? How do you think it compares to smoking? What makes you think that?

23. How addictive do you think e-cigarettes with nicotine are? What makes you think that? What about e-cigarettes without nicotine? Overall, how addictive do you think vaping is compared to smoking? **Probe source and basis of information.**

24. How do you think using e-cigarettes affects people nearby? What makes you think that? Where did you get that information from? **Probe source and basis of information.**

25. How have non-vapers around you reacted when you have been vaping? **Probe: what sorts of things have happened? How did you feel about these? What did you do?**

26. Where do you think people should be allowed to use e-cigarettes? **Probe: Inside their home (where)? Outside their home? Inside workplaces? Inside cars? What about outside places where smoking might not be allowed, such as sports grounds? How about inside places where smoking is not allowed, such as bars and restaurants? Anywhere else you think people should be allowed to use e-cigarettes? Any other places where you think they should not be allowed to use e-cigarettes?**

Information (Ten minutes)
Some countries require e-cigarette manufacturers to provide information on their packaging. I'd like to get your views on some of that information and how useful you think it would be. In particular, I'd like you to think about how a heavily addicted smoker who is thinking of quitting using ECs might respond, and how a young non-smoker who is thinking of experimenting with ECs might respond.
Please look at these messages. Show messages one at a time.

- **E-cigarette liquid containing nicotine is addictive.**
- **To get maximum benefit from e-cigarettes, you must stop smoking completely.**
- **Swallowing nicotine e-liquid is harmful to children.**
- **The long-term effects of using e-cigarettes are not known and may be harmful.**
- **The long-term effects of using e-cigarettes are not known and may be harmful, but are likely to be much less harmful than smoking.**
- **Nicotine overdose may result in headaches, nausea, excessive sweating or difficulty sleeping.**
- **Using e-cigarettes while pregnant may harm your unborn baby**
- **Using e-cigarettes while pregnant may harm your unborn baby, but is likely to be much less harmful than smoking.**

27. For each question, probe **How do you think someone who has smoked for a long time and been unable to quit using other approaches, and who is thinking of using e-cigarettes to try and quit would respond to this message? What about a young person who doesn't smoke but who is thinking of trying e-cigarettes? Is there anything you would do to clarify this message or make it more useful?**

28. **I**s there any information you think should be provided on packaging to help consumers? **Probe responses. Clarify how information could be presented. Where should it be (external pack, internal leaflet, other format)?**

Vaporisers and other substances (Five minutes for this and summary section)

29. Finally, I'd like to ask you whether you've ever used an electronic cigarette or vaporiser with any other substances? What were these? **Probe marijuana.** What made you start vaping these substances rather than smoking them? How did the experience of vaping those substances compare to smoking them?

Summary

30. These are all the questions I wanted to ask you. Do you have any other comments you'd like to make?

31. Finally, I just have a short questionnaire for you to complete. Like the rest of our discussion, the information you provide will be completely confidential and only members of the research team will be able to access it.

    PROVIDE RESPONDENT WITH DEMOGRAPHIC QUESTIONNAIRE AND COLLECT AND CHECK ON COMPLETION

    OFFER KOHA AND COMPLETE RECEIPT CHECKLIST
