# Supplementary material for: A qualitative analysis of electronic nicotine delivery systems (ENDS) uptake and use among young adult never-smokers in New Zealand
Source: PLoS One. 2022 May 27;17(5):e0268449. doi: 10.1371/journal.pone.0268449 (PMC9140280; doi:10.1371/journal.pone.0268449)
Supplement: S2 File — (PDF) [file pone.0268449.s002.pdf]

Human Ethics Committee No. 16/132
October 2016 – October 2017

Supporting informed e-cigarette use: A mixed methods study

Participant Information Sheet

Tēnā koe, Warm Pacific greetings, Hello

We would like to invite you to take part in a study looking at e-cigarette use in New Zealand. Please read this information sheet carefully before deciding whether or not to participate. If you decide to participate, we thank you. If you decide not to take part, there will be no disadvantage to you and we thank you for considering our request.

Who are we?

This study is being led by Professor Janet Hoek. Professor Phil Gendall, Dr Rosalina Richards, Mei-Ling Blank and Dr Lindsay Robertson, are also part of this phase of the project. We are all researchers at the University of Otago in Dunedin.

What is aim of the study?

This project examines how people who have never smoked regularly, but who have started vaping, use e-cigarettes and their perceptions of e-cigarettes and smoking.

Who is being asked to take part?

We want to include diverse participants, both men and women, people of different ethnicities, and different ages, in our study. We would like to invite people who have never smoked regularly and who are now using e-cigarettes at least a couple of times a month to participate. We are looking for people aged between 16 and 65.

Will I be reimbursed for taking part?

We will offer a $40 gift voucher to recognise any costs you incur by participating in the research. You will also be able to receive a copy of your interview transcripts and a summary of the overall research findings, if you wish.

What will be involved in the study?

Should you agree to take part in this project, you will be asked to meet with us for an interview where we will ask you about your smoking and e-cigarette use. The interview will take around an hour. We can arrange for it to take place at a time, and in a location convenient for you. With your permission, we will record the interview so that it can be later transcribed.

We do not anticipate that you will experience any discomfort from participating in the study.

What will I be asked?

We will ask you about your e-cigarette use, and explore what you think about e-cigarettes. If you have ever smoked, we will also ask you to tell us about your smoking. We will explore e-cigarette use over a day or week, and ask you how you plan to use these products in the future. We will ask your thoughts on information messages provided with e-cigarettes in other countries, and any other information you would find helpful.

This project involves an open-questioning technique. We will ask you about smoking and e-cigarettes, but the precise questions have not been set in advance, and will depend on the way the interview develops. The University of Otago Human Ethics Committee has approved the general areas to be explored in the interview.

You will be able to answer any question with as much or as little detail as you like. You may withdraw from the project at any stage without any disadvantage to yourself of any kind.

What other information will be collected and how will it be used?

With your permission, we will also collect the following information:

- Details of your experience with smoking and e-cigarettes, and your thoughts on smoking and e-cigarette use.
- Your age, education, current and past smoking frequency, quit attempts, e-cigarette use and smoking among your immediate family.

Only Professor Janet Hoek, Dr Lindsay Robertson, and Ms Mei-Ling Blank will know all participants' identity. Master lists will be destroyed once the data collection has concluded. Participants will not be identified in any publications.

The results of the project may be published and will be available in the University of Otago Library (Dunedin, New Zealand) but every attempt will be made to preserve your anonymity.

The research has been funded by the Health Research Council of New Zealand, but no commercial use will be made of the data.

How will the information be stored?

Once transcribed and checked, the audio files will be destroyed and the interview transcripts will be kept under password protection. The data collected will be securely stored in such a way that only those mentioned below will be able to gain access to it. Data obtained as a result of the research will be kept for at least five years in secure storage.

Any personal information held on the participants (such as your contact details) will destroyed at the completion of the research. Data from transcribed audio tapes will, in most cases, be kept for much longer or possibly indefinitely.

Other things you need to know

- If we have not already confirmed an interview time, one of our team will contact you in the next few weeks to arrange a time, date and location for the interview.

If you have any questions about our project, either now or in the future, please feel free to contact:

Project Lead

Janet Hoek
Department of Marketing
University Telephone Number: (03) 479 7692
Email Address: janet.hoek@otago.ac.nz

This study has been approved by the University of Otago Human Ethics Committee. If you have any concerns about the ethical conduct of the research you may contact the Committee through the Human Ethics Committee Administrator (ph +643 479 8256 or email gary.witte@otago.ac.nz). Any issues you raise will be treated in confidence and investigated and you will be informed of the outcome.

Human Ethics Committee No. 16/132
October 2016 – October 2017

Supporting informed e-cigarette use: A mixed methods study

CONSENT FORM FOR PARTICIPANTS

I have read the Information Sheet concerning this project and understand what it is about. All my questions have been answered to my satisfaction. I understand that I am free to request further information at any stage.

I know that:-

1. My participation in the project is entirely voluntary;

2. I am free to withdraw from the project at any time without any disadvantage;

3. Personal identifying information (from audio recordings) will be destroyed at the conclusion of the project. Other raw data on which the results of the project depend will be retained in secure storage for at least five years;

4. Aspects of this project will involve an open-questioning technique. The general line of questioning will explore your smoking and e-cigarette use. The precise nature of the questions which will be asked have not been determined in advance, but will depend on the way in which the interview develops and that in the event that the line of questioning develops in such a way that I feel hesitant or uncomfortable I may decline to answer any particular question(s) and/or may withdraw from the project without any disadvantage of any kind.

5. I will receive compensation of $40 to recognise any costs I have incurred by participating in the research.

6. The results of the project may be published and will be available in the University of Otago Library (Dunedin, New Zealand) and every attempt will be made to preserve my anonymity.

I agree to take part in this project.

....
(Signature of participant) (Date)

....
(Printed Name)

…………………………………………………..
Name of person taking consent

This study has been approved by the University of Otago Human Ethics Committee. If you have any concerns about the ethical conduct of the research you may contact the Committee through the Human Ethics Committee Administrator (ph +643 479 8256 or email gary.witte@otago.ac.nz). Any issues you raise will be treated in confidence and investigated and you will be informed of the outcome.
