# Supplementary material for: A qualitative analysis of electronic nicotine delivery systems (ENDS) uptake and use among young adult never-smokers in New Zealand
Source: PLoS One. 2022 May 27;17(5):e0268449. doi: 10.1371/journal.pone.0268449 (PMC9140280; doi:10.1371/journal.pone.0268449)
Supplement: S3 File — (PDF) [file pone.0268449.s003.pdf]

**Theme: Connection and belonging**	**Supporting Quotations**
**Sub-themes**	
Social acceptability	Tom: I enjoyed the Shisha and I enjoyed the Shisha pen. Just inhaling something and I don't know, socially everyone seems to be doing it.<br><br>Libby: I remember last year we were all like, "Ah, vaping sucks. Only weird people do it."... And then this year everyone's like, "I bought a vape," (laughs)
Bonding social capital	Jane: if I'm out with friends, I would tend to vape a lot more as well, because they all enjoy using my vape as well. So they'll like ask me, and then we'll just pass it around.<br><br>Libby: it's either like one vape being passed around, or like multiple vapes being passed around a group of people, or I found just everyone has their own vape (laughs) kind of thing.<br><br>Tom: if it's during the week, when everyone's home 'cause everyone seems to use it in my flat as well….Yeah. If I get it out, then it's the same at parties 'cause no-one really sees them around and yeah, e-everyone seems to pass the thing around all the time, if I ever bring it out….Yeah, they're all using my one, yeah and have ... Yeah. It will just go around in a circle, pretty much. I don't get it back for a while. (chuckles)<br><br>Bailey: your typical scenario where everyone, your friends are socialising again and you want to be involved, and um, the e-cigarettes with nic-, nicotine in them are far being passed around. Um, you'll probably find yourself on them and then all of a sudden addicted to them, sort of thing....Yeah. I'd say that's probably a pathway that I see happening. Not for myself, but other ... Kind of when I talk to friends who are having e-cigarettes and stuff. They'll say that's kind of how they got onto them.
Bridging social capital	Jane: ...a lot of the times people will just ask me if they can try it.<br>**Int:** And, and what do you do when they ask you that?<br>Jane: Um, yeah, I'm fine with it.<br><br>Joseph: Sometimes, I'll have someone else that, that also vapes like want to know uh, what kind of mod I'm using, if I'm blowing out a lot of smoke or something. Because some people like the idea of blowing out a lot of cloud, or you know.<br>Violet: When I talk to people outside of my class, they are like "Oh, that's interesting. Can I try it?"

	Bailey: It's about being able to have my own one.... And, obviously being involved in when everyone goes out for ciggy breaks or e-cigarette breaks on a night out. Go out with them (laughs). Provide for myself, sort of thing.
**Theme: Recognition and social cachet**	
Sub-themes	
Device appearance and extended-self	Iain: I heard about it from a friend and it was one of my mates overseas and it's in Australia and he picked it up and he said you know, it's actually not that bad, it's a bit of fun and yeah, I sort of looked into it and then I think as things started to get a little bit more popular and it became a little bit more socially accepted to have E-cigarettes and all that and they stopped looking so ugly and old fashioned, I was kind of like I might give it a go...<br><br>Jane: .. so we went to this like sort of shisha shop that also sold vapes. And then I thought, "Oh, well, I might just buy a little one for myself," and 'cause, you know, I, I thought it would be a cool thing to have. (laughing)
Performance props and social cachet	Abe: when my friends come down I guess I'll pull it out as a novelty again.<br><br>Joseph: I just thought that it was, that it looked kinda cool, you know, being able to blow smoke out and um, you know, it tastes good. So, I was just like you know, I might try it. Asked him how much it cost and he said $700 for a good third generation one. So, I went out and spent a bit of money on it.<br><br>William: I go out with my friends maybe three, three times a week maybe, um, or over at their flat that's, I take it out. 'Cause they're always quite interested, like they s- I still get a lot of crap for it 'cause it's, 'cause it's vaping. And once they see the tricks they're a lot more into it, and they- they wanna try it. And then like when buying my first, um, vape or- or second, I still got a lot of shit from- from my friends about it. Um, but I guess it-it changes a lot when they realise that I'm not doing it just 'cause I wanna vape, they wanna, I wanna do it 'cause of the tricks and everything.... And so they were very against it first, and then when I bought it and started doing stuff, they were all like that's amazing, I wanna do it now.
Managing stigma	Violet: I guess it feels like I'm doing something that not many people in my demographic do, which is always uncomfortable because then it's kind of like every time you do it, you have to justify it if you are with people. So I tend to do it a lot more at home.... Um, it's not really like, uh, I don't know. Like, I ... I guess I'm not of the

	opinion that, like, everything I do, has to instantly be accepted by the society. Um, so I'm not too hurt by it. It's not like "why won't you embrace my vaping?" Um, it's just mildly annoying but it's the way it is.<br><br>Joseph: Um, saying things like um, you know, hipsters uh, you know. It, it just hipsters, vape or you know, if someone's talking about uh, um, like cold-brew coffee and vaping often go together. So, it's like, you know, having- ... Ah, if an article comes up about coffee, um ... I remember there was one about coffee in Brisbane, where they can, they serve you the three components of the coffee in separate cups and you make your own?... So you get a shot of coffee, some water, and a milk. And of course, it's a deconstructed coffee and it's all the hipster's rave.... So, there are of course comments about um, about, "I'm sure everyone that goes there is a vaper." You know, is vaping, and so on and so forth.<br><br>Bailey: Yeah, it might be just sort of a tradition thing and um, maybe it's not as ... It's getting to be socially accepted. You do see them more often now as moderation goes on. But yeah. I think they kinda just think "Oh, come on just have a real smoke" sort of thing.<br>**Int:And what's not real about an e-cigarette do you think, to these people?**<br>Bailey: Yeah, it must just be the ... They might just know that it's a plastic, think that it's plastic .. Um, so it might just be the physicality of it. Uh, the way it feels.<br><br>Gerry:Because I would never take it out in public.<br>**Int: Mm-hmm (affirmative). What, what made you decide not to take it out in public?**<br>Gerry: I guess the stigma associated with it.<br>**Int: Mm-hmm (affirmative). What, what do you mean by that?**<br>Gerry: I guess it might be viewed as an unpleasant thing to do.<br><br>Iain: I think there's still a lot of stigma...amongst regular smokers about seeing people vape...<br>**Int: And what do you, what's that stigma take the form of?**<br>Iain: Oh it looks gay, it looks stupid, you know what I mean, oh it looks like you're you know, chuffing on a dick, that kind of thing (chuckles)... yeah like it's, it doesn't look cool the way that cigarettes look to a lot of people I guess which is yeah...<br><br>Tara: guess just because there's like a whole, like, "oh, you vape," kind of thing and people judge. Some people, sometimes, I think, yeah**...** Um, I know that there are a lot of like, teenagers, people my age, who are like, "Oh, you vape."

**Theme: Stimulation and engagement**	
Sub-themes	
Flavours	**Int: Were there other parts of the experience that you enjoyed?**<br>Gerry: Um ... Just the flavour, I suppose…. I find it relieves stress.<br>**Int: Mm-hmm (affirmative). How did it relieve stress for you?**<br>Gerry: I guess it's just like when you're stressed and you get lollies. It's kind of that ...<br><br>Iain: if you've got one that's got a terrible flavour, it's almost unusable because it's just, it can leave a really horrible taste in your mouth and one thing that my girlfriend complains about now is if I'm sitting there puffing on it all day like I tend to do sometimes, if the vapour gets a little burnt, it leaves a really bad smell in the room....so it's quite unpleasant if you've got a bad flavour.<br><br>Joseph: I would just go through a bottle each day, just 'cause I enjoyed the flavour, so I'd keep on using it. Um, whereas at least with the nicotine it ... After using it to a, to a point, I will start to feel it and I will start to sometimes even feel ill, so that kinda limits how much I use it.<br><br>Joseph: You can try different flavours, you can ... You know, eh, it's all the flavours, you're, you're not necessarily eating stuff and you can be Puff the Magic Dragon.<br><br>Leonie: I feel like even if I have a favourite now, it changes. You know. Um, I guess at the moment, I have one that tastes like gummies, like specifically a peach flavoured gummy shark that you might get at a dairy, um, and I really like that one, but, I, uh, think my favourite above that might be one that tastes like chocolate milkshakes. Um, and then, also, having one that's minty is really nice, 'cause it's quite refreshing.<br><br>Libby: I just like the fact that it tastes like candy basically… That's what was appealing to me (laughs).<br><br>Violet: there are some [flavours] that's aimed at smokers which is the ones I don't like. The ones that kind of have tobacco flavours. Um, yeah's a lot, there's a ... the ones that are kind of minty, mixed with something else. I really like the more complicated flavours, this one I have is like, uh, cinnamon menthol and mint? Um, and the one that I'm using now Menthol, strawberry, kiwi fruit and pomegranate, I think?
Clouds	Iain: just think it's a very basic and satisfying thing to do just to push out a huge big cloud of something and I really like trying to do you know, smoke rings and that kind of thing with it... and it's just a bit of fun.

	Abe: It ... it just seemed like a novelty of being able to, to play with the smoke [he refers to ENDS aerosol as smoke], do smoke tricks, that sort of stuff. Which is, once again ... another big side of the culture that a lot of people are into.<br><br>Abe: There's nothing I think is pref-preferable about smoking. Um, with vaping I guess, being able to make some sort of fun out of it, with the games, and the tricks, would be one of the only benefits I could think of, over not doing it.<br><br>Iain: I just really like blowing big clouds of vapour....I just think it looks fun...it's different from cigarettes in the sense that you do it to fit in and look like other people, you know what I mean, I just think it's a fun hobby to sit there going (exhales), blowing huge big clouds of vapour, just it's fun...<br><br>**Int: And what is it about getting that smoke or cloud that, that appeals?**<br>Jane: Um, (laughing) I think you just feel a bit cool. (laughing)<br><br>Simon: I think it's [clouds] pretty interesting. It's like some people are going forward with like not just using it for personal, like medicinal use, like more using it for recreational, which is kind of nice. Yeah.<br><br>William: there are always videos going around. More like viral videos that ... people were doing all these tricks. I always thought they were incredibly impressive being able to do stuff like that and it sort of got me hooked into it. Um, so yeah.<br>**Int: So when you said there were always videos going around ... what's this on, like, Facebook? Or** ...<br>William: On Facebook and stuff. Just on my newsfeed. All these social media sites. There were just all these ... check out these vapers who can do all these sort of tricks and sort of ... (Chuckle) I was really interested by it, so I sort of wanted to learn how to do that.
Devices	Joseph: I like to be able to, to be able to change it to what I want. Um, I actually haven't changed it in a long time but that's 'cause I'm really happy with it… But I wouldn't be able to go in and buy a tank and have it run like I've got this running. So, um, the customised, the customisability is quite, quite nice.<br><br>Joseph: the one that got me into it, um, he, he had just gotten into using the RDA's and kinda playing around with making new coils. So, I guess that's where I saw him doing it and then I was, you know, like a little lost duckling. Um, whereas some of my other friends they're just like, "Well, you know, if it smokes, it works."… So, they don't really want to mess around with it…. And um, that's one of the great things about the third generation ones, is you can just kinda go with the tank and you know, be happy with it. Or you can um, get into customising it a lot more.

**Theme: Self-management**	
Sub-themes	
Nicotine and stress management	Gerry: it's like taking deep breaths, as well, while you're doing it, so I guess that's sort of relaxing.<br><br>Iain: Oh it feels relieving...yeah and calming... to smoke [means to vape], yeah. ...it's just a relieving feeling I guess, yeah.<br><br>Jane: once I started doing like vaping myself, I sort of started like noticing the nicotine effects…. Um, like getting a head rush and feeling more relaxed and everything…. So, yeah, after that, I like got really into it. (laughing)<br><br>Jane: It's probably similar to cigarettes like, yeah, you just sort of feel the need to, and you get a little bit like tense and stressed ... and, um, like if I don't have my vape on me when I feel like I need a vape, it's kind of a little bit stressful. (laughing)<br><br>Jane: I'll lie in bed before I go to sleep at night or something, and I'll have a vape .... and it's just sort of like this instant kind of head rush, and then like my whole body just kind of feels very relaxed, and I feel kind of sleepy….And, yeah, just very calm and ...<br><br>Leonie: I'm not really happy with the ambiguity around my health with vaping. But, um ... It sort of tied into the whole coping mechanism thing. Vaping without nicotine, uh, was nice, but it wasn't sort of giving me a, like a rush, or a feeling of relaxation...
Food and drink consumption	Violet: it gives me time to take a break and to, I have a problem with comfort eating a lot, so it's something that I can do instead of just eating all the time like especially, like studying because it's ... I don't know you just have like ups and downs of like stressful time in holidays and you know.<br><br>Iain: I'm quite chubby but I used to be really quite big and having that nicotine intake kind of sated my appetite quite a lot and just having just nicotine without all of the extra crap in cigarettes just made it a lot easier for me to keep control of my weight without having to poison myself with a million other chemicals...<br><br>Simon: talked to my friend and um, just talked to him about how it helps him and what he's doing, and then asked him like if it like helps his eating thing because that was my major thing was eating. It's supposed to help that type of eating process, where I had to stop eating as much or just having something there too. … He's like,

	"Yeah, it helped me like significantly." He's lost a lot of kgs, from one of my friends who doesn't use nicotine um in his, and he told me he had lost some kgs and he was just like it just helps him with eating snacks and stuff, and moving forward from like just eating food constantly. Like, when you're want to binge eat, or you eat something and then you're still kind of hungry, and you're like, "Oh, I'll go grab something more." Then it helped him a lot with it.
**Theme: Rationalisations relative to smoking**	
Sub-themes	
Reduced harms	Leonie: for quite a while, I was having the urge to smoke, and, um, I sort of dealt with that whenever my mental health gets bad. Uh, it's sort of like, my brain wants a coping mechanism, any coping mechanism, you know, and, um, people ... It- it looks as if cigarette smoking might be a helpful one, or something, to my brain. Um, I'm aware of all the risks and all the damages, but I just kept having this like sort of obsessiveness or urge or whatever, like, I want to smoke a cigarette. I feel like that'll make me feel better. Um, so she, uh, was trying to like look out for me, and we- we talked about it a lot, and we did a lot of research, and I decided that, um, the harms were a lot less, and I could start with non-nicotine juice, and that it would give me that sort of act of smoking without any of the nicotine, or any of the, um, the tobacco, uh, toxins or anything like that. So that was, I think, that was mostly what motivated me, is I didn't want to start smoking, even though I wanted to, but I didn't want to, you know?<br><br>Libby: I would 100% say vaping is a lot better, or not better for you, but not as bad for you [as smoking] kind of thing.<br><br>Abe: it seems like they're really pushing for everyone to finish smoking and start smoking e-cigarettes because there's so much more health benefits. Or I wouldn't say benefits, but there's less ... detrimental effects that smoking has.<br><br>Ashley: I think lack of combustion and so less of the, so no carbon monoxide, um, like less of all the other by products.<br><br>Ashley: I guess because (clears throat) I did read up a bit and, um, although they're not completely sure, because of how new it is, in general it did seem less harmful than smoking.<br><br>Dave: there's so many like negative ads about tobacco and smoking. And vaping's seen as like a positive sort of alternative.<br><br>Gerry: Like I said before, I would think that vaping would be better, in terms of health effects compared to

	smoking, just due to the fact that it has two basic ingredients, as opposed to the hundreds of additives that you see in cigarettes, and don't think it would have the same effects on your organ systems and things.<br><br>Leonie: my thoughts are usually about the smell, 'cause, um, cigarette smoke smells so bad that if I were to smoke cigarettes, I wouldn't want to be in a crowd….um, 'cause I would feel real judged, uh, for it, because the smell is so intense, and there's, I think there's a cognitive dissonance as well, because part of how ... Part of feeling judged, if I were to smoke cigarettes is the health effects, and everyone knows that it's really bad for you, um, but for some reason, when I'm vaping, uh, I feel like ... I feel less sort of attached to those health effects, because I don't really know them.<br><br>Bailey: I always ask them for the blueberry (laughs) and um, that's I think, mentally knowing that it was like, healthier, probably helped me lean towards the e-cigarette more than the cigarette.<br><br>Cath: … using an e-cigarette, um, I guess it just has nicotine without all the other stuff added.<br><br>Simon: guess this is a little bit like less harmful for you. Like, um, obviously there's not a lot of major lung cancers, and like tar build ups, and stuff like that.
Easier and more pleasant than smoking	Jane: Like vaping is both describe it as a clean, sort of rush of nicotine. It's like, yeah, very clean. Um, and to them like smoking just feels a bit dirtier, and it's not ... as like smooth maybe.<br><br>Joseph: …compared to smoking, it didn't make me choke as much.<br><br>Leonie: When I was having urges for cigarettes, I went and got a pack at one point, and sat down and smoked a cigarette, and realised that's it way different, and it's not what I wanted, and it's not what I liked, because when you use a vape, it's you breathe in a lot more air, at a time. It's a lot cooler, it's a lot easier to breathe in. Definitely doesn't taste as gross. Um, for some vapes, there's like a hissing noise, so that's kind of different. Um, but, yeah, I think what stands out most to me is how much easier it is to like "take a drag," um, even though I don't know if you are with a vape, but, but yeah. It seems easier, it's more like just breathing instead of pulling on something.<br><br>Libby: if you're vaping, it kind of just tastes like you've eaten something really nice…. Rather than smoking and having bad breath afterwards and stuff like that.
